# Prognostic Relevance of Neutrophil to Lymphocyte Ratio (NLR) in Luminal Breast Cancer: A Retrospective Analysis in the Neoadjuvant Setting

**DOI:** 10.3390/cells10071685

**Published:** 2021-07-03

**Authors:** Antonino Grassadonia, Vincenzo Graziano, Laura Iezzi, Patrizia Vici, Maddalena Barba, Laura Pizzuti, Giuseppe Cicero, Eriseld Krasniqi, Marco Mazzotta, Daniele Marinelli, Antonella Amodio, Clara Natoli, Nicola Tinari

**Affiliations:** 1 Center for Advanced Studies and Technology (CAST), Department of Innovative Technologies in Medicine & Dentistry, G. D’Annunzio University, 66100 Chieti, Italy; laura.iezzi@unich.it (L.I.); natoli@unich.it (C.N.); 2 Cancer Research UK Cambridge Institute, University of Cambridge, Cambridge CB2 0RE, UK; vincenzo.graziano@cruk.cam.ac.uk; 3 Division of Medical Oncology 2, IRCCS Regina Elena National Cancer Institute, 00144 Rome, Italy; patrizia.vici@ifo.gov.it (P.V.); maddalena.barba@ifo.gov.it (M.B.); laura.pizzuti@ifo.gov.it (L.P.); eriseld.krasniqi@ifo.gov.it (E.K.); marcomazzotta88@gmail.com (M.M.); antonella.amodio@ifo.gov.it (A.A.); 4 Department of Surgical, Oncological and Oral Sciences, University of Palermo, 90127 Palermo, Italy; giuseppe.cicero@unipa.it; 5 Oncology Unit, Department of Clinical and Molecular Medicine, Sant’Andrea Hospital, Sapienza University, 00185 Rome, Italy; daniele.marinelli@uniroma1.it; 6 Center for Advanced Studies and Technology (CAST), Department of Medical, Oral and Biotechnological Sciences, G. D’Annunzio University, 66100 Chieti, Italy; ntinari@unich.it

**Keywords:** luminal breast cancer, neoadjuvant chemotherapy, neutrophil to lymphocyte ratio (NLR), predictive/prognostic biomarkers

## Abstract

The neutrophil to lymphocyte ratio (NLR) is a promising predictive and prognostic factor in breast cancer. We investigated its ability to predict disease-free survival (DFS) and overall survival (OS) in patients with luminal A- or luminal B-HER2-negative breast cancer who received neoadjuvant chemotherapy (NACT). Pre-treatment complete blood cell counts from 168 consecutive patients with luminal breast cancer were evaluated to assess NLR. The study population was stratified into NLR^low^ or NLR^high^ according to a cut-off value established by receiving operator curve (ROC) analysis. Data on additional pre- and post-treatment clinical-pathological characteristics were also collected. Kaplan–Meier curves, log-rank tests, and Cox proportional hazards models were used for statistical analyses. Patients with pre-treatment NLR^low^ showed a significantly shorter DFS (HR: 6.97, 95% CI: 1.65–10.55, *p* = 0.002) and OS (HR: 7.79, 95% CI: 1.25–15.07, *p* = 0.021) compared to those with NLR^high^. Non-ductal histology, luminal B subtype, and post-treatment Ki67 ≥ 14% were also associated with worse DFS (*p* = 0.016, *p* = 0.002, and *p* = 0.001, respectively). In a multivariate analysis, luminal B subtype, post-treatment Ki67 ≥ 14%, and NLR^low^ remained independent prognostic factors for DFS, while only post-treatment Ki67 ≥ 14% and NLR^low^ affected OS. The present study provides evidence that pre-treatment NLR^low^ helps identify women at higher risk of recurrence and death among patients affected by luminal breast cancer treated with NACT.

## 1. Introduction

Breast cancer is the second cause of cancer death in women in industrialized countries, despite early diagnoses and therapeutic advances having considerably reduced mortality [1]. Neoadjuvant chemotherapy (NACT) is the standard of treatment in locally advanced breast cancer, but in recent years it has been widely used in operable tumors not only to allow breast-conserving surgery (BCS), but also to test in vivo tumor responsiveness to chemotherapy. This latter aspect is particularly important for triple-negative (TN) or human epidermal growth factor receptor 2 (HER2)-positive breast cancer, since patients who do not achieve a pathological complete response (pCR) following NACT have a dismal prognosis [2,3]. In these cases, further adjuvant chemotherapy can significantly improve long-term outcomes [4,5,6].

This latter strategy is not applicable in patients affected by luminal A- or luminal B-HER2-negative breast cancer (herein referred to as luminal). Indeed, luminal subtypes achieve pCR from NACT infrequently. Still, luminal breast cancers generally maintain a favorable prognosis even in the presence of residual disease [7,8,9,10]. Nonetheless, 6–8% of these patients experiences relapse within 5 years from diagnosis and die due to the disease [11]. Thus, the identification of predictive and prognostic factors in patients with luminal breast cancer candidates to NACT is needed. This would help select those patients at higher risk of recurrence who may benefit from further treatment.

The Neutrophil to Lymphocyte Ratio (NLR) is a peripheral marker of inflammation extensively studied in breast cancer as a potential predictor of response to chemotherapy and long-term outcome. Unfortunately, the evidence emerging from the studies carried out thus far is inconsistent. Indeed, some studies reported an overall worse prognosis for patients with high NLR [12], while others found no evidence in support of the association of interest [13,14], or even opposite results [15].

In the study herein presented, we retrospectively investigated the prognostic impact of pre-treatment NLR in a cohort of 168 patients with luminal breast cancer who received NACT as primary treatment.

## 2. Patients and Methods 

### 2.1. Patients

Patients with early or locally advanced luminal breast cancer who received NACT between January 2004 and December 2019 at the Medical Oncology Units of the “S.S. Annunziata” Hospital of Chieti and at the “G. Bernabeo” Hospital of Ortona were consecutively screened for participation in this study. All conditions that could have affected absolute neutrophil or lymphocyte counts were carefully evaluated. Specifically, patients with autoimmune diseases or infections, as well as those under steroidal, NSAIDs or antibiotic therapy, were excluded from the study.

All breast cancer diagnoses were histologically confirmed. Following NACT, mostly based on the standard regimens containing anthracycline and/or taxanes, all patients underwent surgical procedures as clinically indicated: mastectomy or breast-conserving surgery (BCS) and axillary lymph node dissection or sentinel lymph node biopsy. Adjuvant radiotherapy was administered to patients with BCS as well as to patients who had undergone mastectomy but had stages cT3, cN2 or cN3 at diagnosis or stage pN2 after surgery. All patients received adjuvant hormonal therapy according to current recommendations. The follow-up contacts were carried out at 6-month intervals over the first 5 years and at 12-month intervals thereafter.

Clinical and pathological tumor staging were defined according to the 8th edition of the American Joint Committee Cancer Staging Manual. This study adheres to the REMARK guidelines [16].

### 2.2. Pathological Assessments

All breast cancer biopsies and surgical specimens were processed for immunohistochemistry (IHC) assessment. Tumors were considered estrogen receptor (ER) or progesterone receptor (PR) positive when receptor staining was expressed in at least 10% of cells [17]. Ki-67 was detected by MIB-1 antibody [18] and a cut-off of 14% was set to discriminate between luminal A (<14%) and luminal B (≥14%) tumors [19]. The nuclear grade was assessed according to the Nottingham grading system [20]. HER2 positivity was defined according to the ASCO/CaP guidelines, i.e., a score 3+ in ICH by HercepTest™ (Dako, Milan, Italy) and/or amplification of the inherent gene by FISH or SISH [21]. Only patients diagnosed with ER and/or PR-positive and HER2-negative tumors were included in this study.

Pathological complete response (pCR) was defined as the absence of invasive breast cancer in the breast and axillary lymph nodes in the surgical specimen after NACT (ypT0/ypTis, ypN0). Non-invasive breast residuals (carcinoma in situ) were allowed.

### 2.3. Blood Samples and Data Collection

Peripheral complete blood count was performed at baseline, i.e., immediately before starting NACT. The neutrophil to lymphocyte ratio was provided by the ratio between the absolute count of neutrophils and the absolute count of lymphocytes. All blood cell assessments were centrally performed at our institutional laboratory according to previously established standardized operative procedures.

Data concerning the clinical and pathological features of all patients, along with the type of treatment administered and long-term outcome, were retrospectively collected and entered into an anonymized dedicated database.

### 2.4. Study Endpoint

The main objective of the study was to verify the possible prognostic value of NLR in reference to disease-free survival (DFS) and overall survival (OS). 

### 2.5. Statistical Analysis

The cut-off points for NLR were calculated by the Receiver Operating Characteristic (ROC) curve for the prediction of distant metastasis. The identified cut-off values split our population into NLR^high^ and NLR^low^. The relationships between NLR and key clinical-pathological characteristics were evaluated by Pearson’s χ^2^. 

The Kaplan–Meier method was used to calculate the 10-year rates of DFS and OS in the different patients’ subgroups. The follow-up for OS was defined as the time interval between diagnosis of breast cancer and death, while DFS was intended as the interval between diagnosis and the first appearance of metastatic disease. In patients in whom none of these events occurred, the observational time interval was censored at the last follow-up visit. Differences between curves were evaluated using the log-rank test.

Multivariate analyses were performed using the Cox proportional hazards model according to the backward fitting procedure. Variables with a *p* < 0.10 at univariate analysis were entered in the model. A *p* value of 0.05 or less was considered statistically significant. All statistical analyses were performed using SPSS^®^ software v11.0 (SPSS Inc, Chicago, IL, USA).

## 3. Results

### 3.1. Patient and Tumor Characteristics

We identified 168 patients with luminal breast cancer who had received NACT and with a pre-treatment complete blood cell count reported in our clinical records. Baseline and post-treatment characteristics, overall and across subgroups defined upon NLR cut-off value, are showed in Table 1 and Table 2, respectively.

Median age at diagnosis was 50 years (range: 26–74). Prevalent histology was invasive ductal carcinoma (64.3%), but a relevant number of cases included invasive lobular carcinoma (14.9%) and mixed (ductal/lobular) invasive carcinoma (16.7%). Tumor size at diagnosis was >2 cm (cT2) in the majority of cases (72.6%) and only a few, 2.4% of tumors, were high grade (G3). Based on the Ki67 proliferation index, more than three-quarters of patients (77.4%) had a luminal A tumor subtype, while 22.6% were luminal B-HER2-negative breast cancers. One hundred and thirty-seven patients (81.5%) were treated with a classical anthracycline- and taxane-based sequential chemotherapy, and most patients (87.5%) received at least four cycles of chemotherapy.

After NACT, 99 patients (58.9%) underwent a conservative surgical approach, while the remaining 69 (41.1%) were treated by mastectomy (Table 2). Only 16 (9.5%) patients obtained a pCR (10 luminal B and 6 luminal A). The post-treatment Ki67 index in the 152 cases with residual tumor was ≥14% in 12 (8.4%) patients as a result of a change from luminal B to luminal A in 19 patients (50% of 38 initially luminal B) by effect of NACT, and the conversion of three luminal A to luminal B. Residual disease in breast was <2 cm (ypT0 or ypT1) in 111 (66.1%) patients and 127 (75.6%) had fewer than three positive axillary lymph nodes (ypN0 or ypN1). Post-surgery stage was 0 or I in 47 (28.0%) patients.

### 3.2. Relationship between Clinical-Pathological Characteristics and NLR

In our population, the median value of neutrophils was 3820/μL (range: 1310–8830), while that of lymphocytes was 1920/μL (range: 700–6020). No patient had neutropenia (<1000/μL) and only three patients had lymphocytosis (>4000/μL).

According to the ROC analysis, the best cut-off values of NLR to identify patients at higher risk of recurrence were <2.12 (AUC: 0.645, 95% CI: 0.57–0.72, *p* = 0.021). This cut-off had a sensitivity of 88.9% and a specificity of 49.3%. The NLR distribution according to basal and post-treatment clinical-pathological characteristics of patients is reported in Table 1 and Table 2, respectively.

Compared to ductal invasive carcinoma, non-ductal (lobular or mixed) histology was significantly associated with NLR < 2.12 (NLR^low^) (*p* = 0.012). None of the other variables analyzed were significantly associated with NLR. In more detail, no association was observed with pCR.

### 3.3. Long-Term Outcome

After a median follow-up of 7.98 years (range: 1.05–15.25), 18 (10.7%) patients developed distant metastases (10 liver and/or lung, 5 bone only, and 3 brain) and 10 (6.0%) patients had died.

Results of univariate analysis of clinical-pathological characteristics associated with DFS and OS, including NLR, neutrophil count and lymphocyte count, are shown in Table 3.

**Figure 1:**
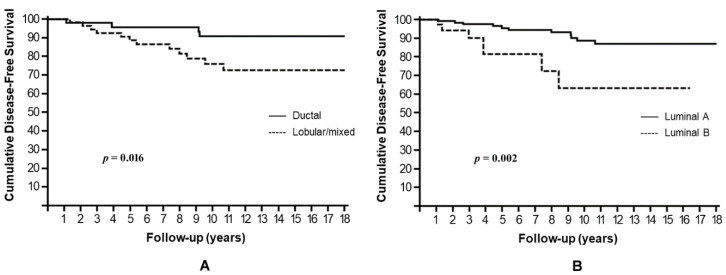
Cumulative disease-free survival stratified by histology (**A**) and molecular subtype (**B**).

**Figure 2:**
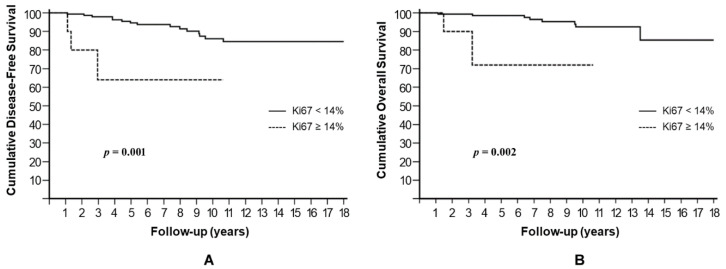
Cumulative disease-free survival (**A**) and overall survival (**B**) stratified by post-treatment Ki67 index.

Non-ductal histology, luminal B subtype, and Ki67 ≥ 14% in residual tumor after NAC were the factors associated with a significantly worse DFS. In Kaplan–Meier analysis, the estimated cumulative 10-year DFS rates were 76.1% for non-ductal tumors compared to 90.8% for their ductal counterparts (HR: 3.12, 95% CI: 1.24–8.28, *p* = 0.016) (Figure 1A); 63.4% for luminal B compared to 88.8% for luminal A (HR: 3.81, 95% CI: 2.04–29.12, *p* = 0.002) (Figure 1B); and 64% for Ki67 ≥ 14% compared to 86.1% for Ki67 < 14% (HR: 7.13, 95% CI: 5.26–100, *p* = 0.001) (Figure 2A). A trend towards a shorter DFS was observed in patients who underwent mastectomy, compared to those treated with BCS (*p* = 0.058), and in patients with pathological stage II or III after NACT, compared to those with stages 0–I (*p* = 0.070).

Ki67 ≥ 14% in residual tumor was also significantly associated with lower 10-year OS rates (64.1% vs. 86%, *p* = 0.002) (Figure 2B). A trend towards worse OS was observed for non-ductal histology (*p* = 0.069) as well as for mastectomy (*p* = 0.063), while the luminal B subtype did not affect OS significantly (*p* = 0.118).

**Figure 3:**
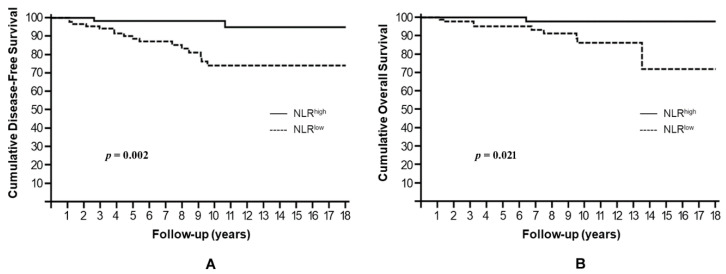
Cumulative disease-free survival (**A**) and overall survival (**B**) stratified by NLR.

NLR^low^ resulted significantly associated with higher risk of disease recurrence and death, showing a 10-year DFS rate of 74.0% compared to 98.3% for NLR^high^ (HR: 6.97, 95% CI: 1.65–10.55, *p* = 0.002) (Figure 3A) and a 10-year OS rate of 86.2% compared to 97.9% for NLR^high^ (HR: 7.79, 95% CI: 1.25–15.07, *p* = 0.021) (Figure 3B).

**Figure 4:**
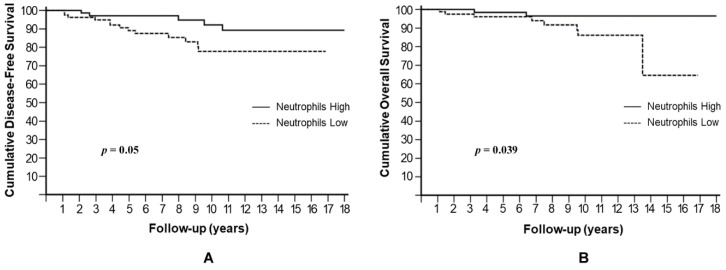
Cumulative disease-free survival (**A**) and overall survival (**B**) stratified by absolute neutrophil number.

Both an absolute neutrophil number below the median value, i.e., <3820/μL, or an absolute lymphocyte number above the median value, i.e., >1920/μL, would have contributed to NLR^low^. Therefore, we separately analyzed neutrophils and lymphocytes for their possible influence on patients’ prognoses. A low neutrophil level was significantly associated with higher risk of metastases and death, with a 10-year DFS rate of 77.8% compared to 92.2% for high neutrophils (HR: 2.51, 95% CI: 1.00–6.34, *p* = 0.05) (Figure 4A) and a 10-year OS rate of 86.1% compared to 96.5% for high neutrophils (HR: 3.73, 95% CI: 1.06–12.99, *p* = 0.039) (Figure 4B). 

**Figure 5:**
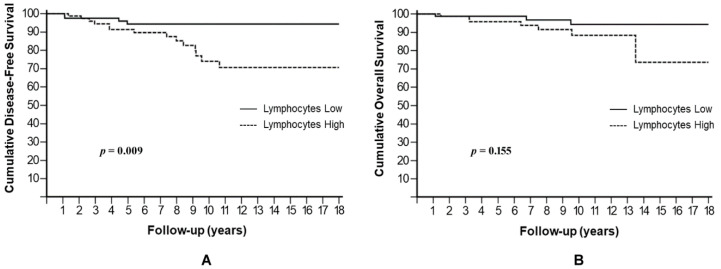
Cumulative disease-free survival (**A**) and overall survival (**B**) stratified by absolute lymphocyte number.

Similarly, a high lymphocyte level showed a significantly reduced 10-year DFS rate of 74% compared to 94.4% for low lymphocytes (HR: 3.45, 95% CI: 1.37–8.74, *p* = 0.009) (Figure 5A), but it not reached statistical significance in OS (*p* = 0.155) (Figure 5B).

In multivariate analysis, the luminal B subtype (*p* = 0.049), Ki67 ≥ 14% in residual tumor (*p* = 0.024), and NLR^low^ (*p* = 0.033) were independent prognostic factors for DFS, while only Ki67 ≥ 14% (*p* = 0.024) and NLR^low^ (*p* = 0.042) maintained significance for OS (Table 4).

## 4. Discussion

In this retrospective study, we examined the prognostic role of pre-treatment NLR in a cohort of 168 early or locally advanced breast cancer patients with luminal tumor treated with NACT. We found that NLR^low^ was associated with adverse long-term outcome in reference to DFS and OS. 

Furthermore, we found that DFS was affected by non-ductal (lobular or mixed) histology, by luminal B subtype, and by Ki67 ≥ 14% in residual tumor after NACT. These results are in line with expectations. In fact, a non-ductal histology, in particular lobular invasive carcinoma, predicts a poor response to NACT [22,23,24] and a shorter survival [25] compared to ductal tumors. Similarly, luminal B breast cancer, defined by Ki67 ≥ 14%, is a well-recognized subtype with worse prognosis compared to luminal A [26,27], and patients with high post-treatment Ki67 levels have been shown to be at higher risk of recurrence and death compared with patients with low Ki67 levels [28].

In multivariate analysis, non-ductal histology was no longer significant, while the prognostic role of NLR^low^, luminal B, and post-treatment Ki67 ≥ 14% was maintained. This latter result can be explained by the significant correlation of non-ductal histology with NLR^low^ (*p* = 0.012). Consistently with previous studies [9,29], a trend towards shorter DFS was observed for patients who underwent mastectomy (vs. BCS) and for those with more advanced stage of disease after surgery (stages II–III vs. stages 0–I), parameters directly linked to lack of response to NACT.

NLR^low^ and post-treatment Ki67 ≥ 14% were also factors that negatively influenced OS (*p* = 0.01 and *p* = 0.002, respectively), along with the necessity to perform mastectomy after NACT and non-ductal histology, characteristics that in our population were associated with a trend towards significance (*p* = 0.068 and *p* = 0.069, respectively). In multivariate analysis, only NLR^low^ and post-treatment Ki67 ≥ 14% were significantly associated with shorter OS.

To our knowledge, this is the first study showing an adverse prognostic effect of NLR^low^ in a subgroup of breast cancer patients. NLR has been widely studied as a marker of the host systemic inflammatory response during cancer development and progression and its elevation is associated with poor prognosis in several cancers, including breast cancer [30]. Its prognostic role has been well defined in more advanced stage of disease, where the boosted inflammatory response, usually revealed by increased level of C-reactive protein and hypoalbuminemia, can promote tumor growth through the production of cytokines and growth factors [31,32].

In breast cancer, several studies have investigated NLR as a prognostic factor in the adjuvant setting. Most of them did not differentiate among breast cancer subtypes and a general correlation of NLR^high^ with worse survival has been reported [33]. Interestingly, a recent meta-analysis analyzed NLR in the different breast cancer subtypes and found an association between NLR^high^ and OS only for HER2-positive and TN tumors, but not for luminal A or luminal B cancers [34]. This may be indicative of a different biological behavior of these breast cancer subtypes with respect to the systemic inflammatory response.

Few studies have investigated pre-treatment NLR as predictive/prognostic factor in patients treated with NACT. This setting offers the chance to assess the role of NLR in the response to treatment, and, more specifically, its association with pCR. We have previously described higher pCR rates in patients with NLR^low^ compared to those with NLR^high^ in a population including all breast cancer subtypes [35]. Similarly, a further study showed an increased pCR rate in the group of patients with NLR^low^, but exclusively in TN tumor [36]. However, other studies failed to demonstrate any association between NLR and pCR [37,38].

Inconsistent results have also been reported for long-term outcome after NACT. Some studies showed an association of NLR^high^ with shorter survival [39,40], while others found no prognostic correlations [37,38,41]. Among these studies, which included all breast cancer molecular subtypes, only one single study performed a subgroup analysis showing that NLR^high^ was associated with shorter DFS and OS in patients with TN tumors who achieved pCR, but not in luminal subtypes [41]. Conversely, Koh et al. reported that NLR^high^ was an independent prognostic factor in a group of 167 patients with luminal HER2-negative breast cancer [42]. A recently published study on a large cohort of breast cancer patients (1519 cases) treated with NACT and stratified by molecular subtype (261 TN, 377 HER2-positive, and 881 luminal-HER2-negative) found that pre-treatment NLR^high^ was independently associated with a worse OS in TN and HER2-positive breast cancer, but no association was observed in luminal tumors [43].

Taken together, with the exception of the Koh’s study, the prognostic value of NLR in early breast cancer seems to be driven by the molecular subtype, although the number of studies addressing this issue is currently limited. The available evidence points to an adverse prognostic effect of NLR^high^ limitedly to the subgroups of patients with TN or HER2-positive tumors.

In our study we focused on luminal subtype and found the opposite of what has been reported for TN or HER2-positive tumors, i.e., NLR^low^, rather than NLR^high^, was associated with shorter survival. We also showed that both elevation in lymphocytes and/or reduction in neutrophils were responsible for the negative long-term outcome associated with NLR^low^. However, in our population, no patient had neutropenia (<1000/μL) and only three patients had lymphocytosis (>4000/μL). Thus, low neutrophils and high lymphocytes were just defined with respect to their median values used as a cut-off, but still remained within the limits of normal range. For this reason, along with its higher statistical power, NLR^low^ was a more appropriate parameter for multivariate analysis. In the following lines, we attempt to provide explanations for the adverse prognostic role of NLR^low^ in luminal breast cancer, considering both lymphocyte elevation and neutrophil reduction.

It is noteworthy that breast cancer subtypes greatly differ not only by ER, PR or HER2 expression, but also by tumor mutation burden and tumor microenvironment. The tumor mutation burden reflects the amount of tumor somatic mutations and the higher this level, the higher the chances that new antigens are recognized as non-self and trigger an immune response against cancer [44,45]. Breast cancer has an intermediate level of tumor mutation burden compared to other types of cancers [46], which is higher in TN and HER2-positive tumor compared to luminal tumor [47]. In addition, the tumor microenvironment is now recognized as a pivotal regulator of the immune response against cancer [48] and it is clearly influenced by the specific molecular subtype of breast cancer [49]. It has been reported that tumor-infiltrating lymphocytes (TILs) are more frequently observed in TN or HER2-positive breast cancer [50], and higher levels in tumor stroma are associated with higher rate of pCR [51] and better prognosis [52]. On the contrary, in luminal tumors, the degree of TILs has the opposite prognostic meaning, i.e., higher levels of TILs are associated with poorer prognosis [52]. At the time of writing this manuscript, the underlining mechanisms to this finding are not fully understood. It is conceivable that the lymphocyte infiltrate of HER2-positive or TN subtypes is different from that of luminal tumors, or that hormones negatively modulate the tumor-associated immunological cells. Another possibility is that immune cell activation may affect responses to hormone therapy [53,54].

The contradictory results of TILs across the different breast cancer subtypes resemble what we have observed for NLR in the present study. Differently from HER2-positive or TN tumors, in luminal breast cancer NLR^low^ is an adverse prognostic factor for survival, suggesting a different immune regulation in this tumor subtype. Interestingly, NLR may reflect the immune cell infiltrate of tumor stroma and inversely correlate with TILs, i.e., the higher the TIL level, the lower NLR [55,56,57,58]. An inverse correlation has also been found between absolute neutrophil count and TILs in breast cancer [59]. Thus, we could speculate that in luminal tumors higher TILs are associated with lower NLR and this condition affects immune response and patients’ prognoses. 

However, a low NLR may also be dependent on neutrophil number reduction. While a different function of TILs across the different breast cancer subtypes has been demonstrated, as described above, very little is known about the role and activation of neutrophils. Neutrophils are emerging as major players in defining the fate of cancer development, promoting tumor growth and progression towards a metastatic disease or favoring killing of tumor cells and cancer regression [60]. The first scenario has been highlighted by a meta-analysis of the association between tumor-associated neutrophils (TANs) and prognosis in different cancer types [61]. In this study, the presence of TANs in tumor tissue was predictive of worse DSF and OS [61]. Interestingly, TANs are abundant in the microenvironment of TN and HER2-positive breast cancer and may therefore contribute to the aggressiveness and poor prognosis of these subtypes [62]. However, the presence of TANs is not sufficient, by itself, to define a pro-tumorigenic effect. In fact, a positive prognostic role of TANs has also been described [63]. It is now recognized that neutrophils can be polarized by the tumor microenvironment toward an anti-tumor (N1) or a pro-tumor (N2) phenotype, depending on the different cytokine and chemokine milieu [64]. The former is mainly determined by exposure of neutrophils to type-I interferon [65]; the latter is prompted by transforming growth factor (TGF)-β [64,66,67] and granulocyte colony-stimulating factor (G-CSF) [68]. N1 neutrophils exert an anti-tumor effect through the production of hypochlorous acid (HOCl), reactive oxygen species (ROS), tumor necrosis factor (TNF)-α, and nitric oxide (NO) and promote an immunogenic microenvironment by inhibiting interleukin (IL)-17 production and exhibiting increased antibody-dependent cell cytotoxicity (ADCC) [60]. To the contrary, N2 neutrophils support tumor growth through the production of vascular endothelial growth factor (VEGF), IL-1β, IL-6, and IL-17, promote an immune-suppressive microenvironment by recruiting Treg, and favor the metastatic process by inducing neutrophil extracellular trap (NET) formation [60], a type of neutrophil death leading to the release of DNA–histone complexes and proteins to form net-like structures.

The evidence that increased levels of neutrophils (both peripheral and in primary tumors) in triple-negative and HER2-positive tumors are associated with tumor progression and dismal prognosis is indicative of a prevalent N2 phenotype in these tumors. Consistently, overexpression of N2-promoting cytokines, TGF-β and G-CSF, as well as NET formation, have been frequently observed in TN breast cancer [69,70,71]. Due to the lack of information on neutrophil characterization in breast cancer subtypes in previous publications, we can try to explain the association between low neutrophils and poor prognosis in patients with luminal breast cancer assuming that the tumor microenvironment of luminal tumors may induce neutrophil polarization toward the pro-immunogenic N1 phenotype. The presence of an anti-tumor microenvironment associated with a more favorable prognosis has been described in luminal tumors [49]. Given these circumstances, a decrease in neutrophils determines a reduction in N1-dependent anti-tumor activity, thus promoting tumor immune escape and progression. Another possibility is that even luminal tumors polarize neutrophils toward the N2 phenotype, but they are unable to mobilize and recruit large numbers of neutrophils, as triple-negative and HER2-positive tumors do. As a consequence, the induction of NET formation leads to a decreased number of neutrophils. In this case, a low level of neutrophils is a marker of high NET formation and, therefore, of cancer progression. This hypothesis is supported by two clues: i) TANs are scarcely present in luminal tumors [62]; ii) the microenvironment of luminal tumors may exhibit immune suppressive properties characterized by the presence of M2-like macrophages and TGF-β pathway activation [72,73], and an increase in NET formation has been observed in response to TGF-β [74].

Further studies on the association between TILs/TANs and NLR in breast cancer and on the characterization of the immune cell infiltrate in tumor microenvironment are needed to clarify the observed discrepancy between luminal and non-luminal tumors. Different T lymphocyte populations, including CD4^+^, CD8^+^, and Treg, along with different neutrophil phenotypes (N1 or N2), may be responsible for the balance between pro-inflammatory and pro-immunogenic responses in the different breast cancer subtypes, and this may eventually influence clinical outcome.

The finding of our study should be interpreted with caution due to its retrospective design and the relatively limited sample size. In addition, we did not have information about basal level of LDH, C-reactive protein, and albumin, parameters that could be helpful for the interpretation of NLR levels in the context of the inflammatory status of the patients. However, the present study supports the emerging evidence of a diverse immune microenvironment in the different breast cancer subtypes and proposes a possible role of NLR in breast cancer as an indicator of activity of the immune system against cancer, rather than a mere marker of the host’s systemic inflammation. 

## 5. Conclusions

We suggest that NLR^low^ may be an indicator of inadequate anti-cancer immune response and, therefore, of dismal long-term prognosis in patients with luminal breast cancer treated with NACT.

## Figures and Tables

**Table 1:** Pre-treatment characteristics of the study patients for the overall cohort and by NLR.

Variable	*n* (%) (*n* = 168)	NLR		
		Low (%) (*n* = 92)	High (%) (*n* = 76)	*p* Value
**Median Age, Years (Range)**	50 (26–74)			
**Age (Years)**				0.057
≤50	87 (51.8)	41 (44.6)	46 (60.5)	
>50	81 (49.2)	51 (55.4)	30 (39.5)	
**Histologic Type**				0.012
Ductal	108 (64.3)	53 (57.6)	55 (72.4)	
Lobular	24 (14.9)	14 (15.2)	10 (13.2)	
Ductal/lobular	28 (16.7)	17 (18.5)	11 (14.5)	
Others	8 (4.10)	8 (8.70)	0 (0.00)	
**Grade**				0.303
G1	82 (48.8)	47 (51.1)	35 (46.1)	
G2	62 (36.9)	30 (32.6)	32 (42.1)	
G3	4 (2.40)	3 (3.30)	1 (1.30)	
Unknown *	20 (11.9)	12 (13.0)	8 (10.5)	
**Clinical T**				0.087
cT1	14 (8.30)	5 (5.40)	9 (11.8)	
cT2	122 (72.6)	72 (78.3)	50 (65.8)	
cT3	26 (15.5)	13 (14.1)	13 (17.1)	
cT4	6 (3.60)	2 (2.20)	4 (5.30)	
**Molecular Subtype**				0.171
Luminal A	130 (77.4)	67 (72.8)	63 (82.9)	
Luminal B/HER2-	38 (22.6)	25 (27.2)	13 (17.1)	
**Type of NACT**				
EC	25 (14.9)	12 (13.0)	13 (17.1)	0.201
EC-T	137 (81.5)	75 (81.5)	62 (81.6)	
Others	6 (3.60)	5 (5.50)	1 (1.30)	
**No. of NACT Cycles**				
≤4	21 (12.5)	11 (12.0)	10 (13.2)	1.000
>4	147 (87.5)	81 (88.0)	66 (86.8)	

* Unknown cases were not included in the analysis. NACT, neoadjuvant chemotherapy; EC, epirubicin and cyclophosphamide; T, taxane.

**Table 2:** Post-treatment characteristics of the study patients for the overall cohort and by NLR.

Variable	*n* (%) (*n* = 168)	NLR		
		Low (%) (*n* = 92)	High (%) (*n* = 76)	*p* Value
**Type of Surgery**				0.519
BCS	99 (58.9)	57 (62.0)	42 (55.3)	
Mastectomy	69 (41.1)	35 (38.0)	34 (44.7)	
**pCR**				0.890
Yes	16 (9.50)	9 (9.80)	7 (9.20)	
No	152 (90.5)	83 (90.2)	69 (90.8)	
**Ki67 in Residual Tumor**				0.999
<14%	140 (83.4)	77 (91.6)	63 (92.6)	
≥14%	12 (7.10)	7 (8.40)	5 (7.40)	
Not determinable	16 (9.50)			
**Size of Residual Tumor**				
≤2 cm	111 (66.1)	59 (64.1)	52 (68.4)	0.674
>2 cm	57 (33.9)	33 (35.9)	24 (31.6)	
**No. of Metastatic Nodes**				
≤3	127 (75.6)	74 (80.4)	53 (69.7)	0.154
>3	41 (24.4)	18 (19.6)	23 (30.3)	
**Stage**				
0–I	47 (28.0)	25 (27.2)	22 (28.9)	0.472
II	75 (44.6)	46 (50.0)	29 (38.2)	
III	46 (27.4)	21 (22.8)	25 (32.9)	

BCS, breast-conserving surgery; pCR, pathological complete response.

**Table 3:** Univariate analysis of clinical-pathological factors predictive of 10-year DFS and OS.

Variable	n	DFS			OS		
		10-Year (%) *	HR (95% CI)	*p*-Value	10-Year (%) *	HR (95% CI)	*p*-Value
**Age at Diagnosis (Year)**							
≤50	87	89.1	1.00		92.0	1.00	
>50	81	79.9	0.55 (0.22–1.4)	0.213	90.9	0.57 (0.16–1.98)	0.376
**Histological Type**							
Ductal	108	90.8	1.00		96.5	1.00	
Lobular or mixed	52	76.1	3.12 (1.24–8.28)	0.016	84.6	3.24 (0.91–11.38)	0.069
**Molecular Subtype**							
Luminal A	130	88.8	1.00		92.5	1.00	
Luminal B/HER2-	38	63.4	3.81 (2.04–29.12)	0.002	89.9	2.87 (0.69–27.33)	0.118
**Grade**							
G1	80	81.2	1.00		91.8	1.00	
G2-G3	66	92.1	1.50 (0.51–4.25)	0.482	95.8	1.55 (0.33–7.17)	0.590
**Type of Surgery**							
BCS	99	88.9	1.00		83.6	1.00	
Mastectomy	69	78.6	2.43 (0.96–6.44)	0.058	97.0	3.33 (0.94–11.8)	0.063
**pCR**							
Yes	16	90.0	1.00		90.0	1.00	
No	152	84.2	2.33 (0.45–7.66)	0.396	91.8	1.10 (0.15–8.16)	0.930
**Ki67 in Residual Tumor**							
<14%	140	86.1	1.00		92.5	1.00	
≥14%	12	64.0	7.13 (5.26–100)	0.001	72.0	31.0 (8.41–100)	0.002
**Size of Residual Tumor**							
≤2 cm	111	87.8	1.00		92.0	1.00	
>2 cm	57	78.5	2.03 (0.81–5.77)	0.125	90.4	1.29 (0.35–4.85)	0.691
**No. of Metastatic Nodes**							
≤3	127	85.0	1.00		91.8	1.00	
>3	41	84.4	1.48 (0.49–4.86)	0.453	90.3	1.51 (0.35–7.19)	0.545
**Stage**							
0–I	47	93.6	1.00		93.6	1.00	
II-III	121	81.0	2.52 (0.93–6.87)	0.070	90.5	1.52 (0.38–6.12)	0.347
**NLR**							
High	76	98.3	1.00		97.9	1.00	
Low	92	74.0	6.97 (1.65–10.55)	0.002	86.2	7.79 (1.25–15.07)	0.021
Neutrophils **							
High	84	92.2	1.00		96.5	1.00	
Low	84	77.8	2.51 (1.00–6.34)	0.050	86.1	3.73 (1.06–12.99)	0.039
Lymphocytes **							
High	84	94.4	1.00		94.3	1.00	
Low	84	74.0	3.45 (1.37–8.74)	0.009	88.4	2.46 (0.71–8.54)	0.155

* Unadjusted Kaplan–Meier estimates. ** Stratified using the median value of absolute count as cut-off. BCS, breast-conserving surgery; pCR, pathological complete response.

**Table 4:** Multivariate analysis of factors influencing DFS and OS.

Disease-Free Survival	HR (95% CI)	*p*-Value
**Histological Type** Non-ductal vs. Ductal	1.90 (0.67–5.44)	0.228
**Molecular Subtype** Luminal B vs. Luminal A	3.00 (1.00–9.84)	0.049
**Type of Surgery** Mastectomy vs. BCS	1.96 (0.72–5.38)	0.188
**Ki67 in Residual Tumor** ≥14% vs. <14%	6.32 (1.27–31.29)	0.024
**Stage** II–III vs. 0–I	4.52 (0.91–22.42)	0.064
**Peripheral Markers of Inflammation** NLR^low^ vs. NLR^high^	5.36 (1.14–25.17)	0.033
**Overall Survival**		
**Histological Type** Non-ductal vs. Ductal	2.08 (0.46–9.34)	0.337
**Type of Surgery** Mastectomy vs. BCS	2.55 (0.59–10.91)	0.187
**Ki67 in Residual Tumor** ≥14% vs. <14%	7.27 (1.29–40.68)	0.024
**Peripheral Markers of Inflammation** NLR^low^ vs. NLR^high^	8.90 (1.08–73.39)	0.042

BCS, breast-conserving surgery.

## Data Availability

Not applicable.
